# A generalized Poisson regression analysis of determinants of early neonatal mortality in Ethiopia using 2019 Ethiopian mini demographic health survey

**DOI:** 10.1038/s41598-024-53332-5

**Published:** 2024-02-02

**Authors:** Fekadeselassie Belege Getaneh, Alemu Gedefie Belete, Aznamariam Ayres, Tewoflos Ayalew, Amare Muche, Lemma Derseh

**Affiliations:** 1 College of Medicine and Health Sciences, Wollo University, P.O. box 1145, Dessie, Ethiopia; 2 College of Medicine and Health Sciences, University of Gondor, Gondor, Ethiopia

**Keywords:** Health care, Medical research

## Abstract

Neonatal mortality within the first few days of life is a pressing issue in sub-Saharan Africa, including Ethiopia. Despite efforts to achieve the targets set by the Sustainable Development Goals, the rate of neonatal mortality in Ethiopia has increased from 29 to 33 deaths per 1000 live births. This study aimed to investigate and identify significant determinants of neonatal mortality within the first 72 h of life in Ethiopia. Utilizing data from the 2019 Ethiopia Demographic and Health Survey, we employed Generalized Poisson regression analysis following rigorous model fitness assessment. Our study encompassed 5527 weighted live-born neonates. Among women in their reproductive years, 3.1% (n = 174) experienced at least one very early neonatal death. Multiple births (Incidence Risk Ratio (IRR) = 3.48; CI = 1.76, 6.887) and birth order six or above (IRR = 2.23; CI = 1.008, 4.916) were associated with an increased risk of neonatal death within the first 72 h. Conversely, household size (IRR = 0.72; CI = 0.586, 0.885) and additional feeding practices (IRR = 0.33; CI = 0.188, 0.579) were found to mitigate the risk of very early neonatal mortality per mother in Ethiopia. Interventions targeting the identified risk factors and promoting protective factors can contribute to reducing very early neonatal mortality rates and improving the well-being of mothers and their newborns. Further research and implementation of evidence-based strategies are needed to address these challenges and ensure better neonatal outcomes in Ethiopia.

## Introduction

Mortality in the neonatal period, spanning the first 28 days of life, is a crucial indicator of a population’s overall health and well-being, reflecting its socioeconomic status^1^. Despite significant global declines in neonatal mortality over the past few decades, developing countries continue to face substantial challenges in this area. In 2020, an estimated 5.0 million children under the age of five and 2.4 million infants lost their lives, with newborns accounting for half of these fatalities^2^. This alarming reality underscores the urgent need for research and intervention to address neonatal mortality, attracting the attention of academics, policymakers, and public health practitioners worldwide^3^.

Among the Sustainable Development Goals (SDGs) set forth by the United Nations is a specific target to reduce the neonatal mortality rate to 25 per 1000 live births by 2030^4^. Achieving this ambitious goal hinges on effectively addressing neonatal mortality in high-risk countries, particularly in developing nations where 98% of neonatal deaths occur^2^.

Ethiopia, the second most populous nation in Africa, has witnessed a decline in both neonatal and under-five mortality rates. However, significant regional disparities persist, potentially attributed to variations in cultural practices, socioeconomic levels, and environmental conditions^5,6^.

Studies have consistently shown that approximately three-quarters of neonatal deaths occur within the first week of life, known as the early neonatal period^7–9^. This critical phase emphasizes the importance of concentrated efforts to improve neonatal survival during this vulnerable period. A concerning reality is that many neonatal deaths in underserved communities remain unrecorded or unregistered, as parents may not seek timely medical attention due to fear or lack of access to healthcare facilities^10^.

While researchers have dedicated efforts to understanding the causes of neonatal and perinatal mortality in Ethiopia, studies have largely overlooked a crucial aspect for policymakers: the distinct circumstances surrounding neonatal deaths within the first 72 h of life. This gap in knowledge hinders effective intervention strategies.

Our study addresses this critical gap by employing count regression models to analyze the impact of various factors on neonatal mortality within the first 72 h of life in Ethiopia. By identifying the individual risk factors associated with mortality, we aim to inform targeted interventions and improve neonatal survival outcomes.

## Methods

### Data source and preparation

This study utilized data from the 2019 Ethiopian Demographic and Health Survey (EDHS), the country’s second Mini Demographic and Health Survey (EMDHS) and fifth DHS overall. The survey, conducted by the Ethiopian Public Health Institute (EPHI) in collaboration with the Federal Ministry of Health, the Central Statistical Agency (CSA), and ICF, employed a nationally representative sample of 8663 households, or 8855 women of reproductive age (aged 15 to 49). Data collection took place between March 21 and June 28, 2019, generating estimates for both urban and rural areas, as well as at the national and regional levels. Comprehensive data on respondents’ background characteristics, fertility factors, marriage, family planning knowledge and practices, child feeding customs, children’s nutritional status, childhood mortality, and height and weight of infants aged 0–59 months were collected^5^. This study includes full survey results for Ethiopia’s nine regional states, two municipal administrations, and the country as a whole. The DHS website (http://dhsprogram.com) provides access to the datasets used in this study.

Study sample for this analysis, a total weighted sample of 5527 completed individual responses from mothers within the reproductive age group who were interviewed about neonatal deaths (deaths of newly born babies within the first 72 h of age) occurring in the preceding five years before the survey was included. Finally, the Ethiopian Public Health Institute’s rules and recommendations were rigorously followed for all methodological components used in this study.

### Variables in the study

The dependent variable (Yi) for this study is the number of newborn infant deaths within the first 72 h of life that each mother has encountered throughout her reproductive life, and it is assumed to have values of 0, 1, 2, 3…. Explanatory variables (i.e., mortality determinants) are listed in Table 1 along with their definitions and categories (Table 1).

**Table 1:** List of explanatory variables with their classification.

S. no	List of variables	Description
1	Respondents current age	Continuous data
2	Marital status	0 = Married
		1 = Unmarried
3	Educational status	0 = technical or/higher education
		1 = not educated
		2 = Primary education
		3 = secondary education
4	Wealth index	0 = above average
		1 = below average
		2 = average
5	Region	0 = Urban cities (Addis Ababa, Dire Dawa and Harari)
		1 = Agricultural (Tigray, Amhara and Oromia)
		2 = Pastoralists (Afar, Somali, Benishangul Gumuz, SNNPR and Gambela)
6	Place of residence	0 = urban and 1 = rural
7	ANC follow-up	0 = four +
		1 = one to three
		2 = No ANC visit
8	Risk fertility behavior	0 = No
		1 = Yes
9	Place of delivery	0 = public health facilities
		1 = home delivery
		2 = private institutions
10	C/S delivery	0 = No
		1 = Yes
11	Birth order	0 = 2nd to 5th
		1 = first
		2 = sixth and above
12	Birth spacing	0 = 24–36 month
		1 = < 24 months
		2 = > 36 months
13	Newborn danger sign counseling	0 = Yes
		1 = No
14	Additional feeding	0 = No
		1 = Yes
15	Month of birth	0 = Bega (October to January)
		1 = Belg (February to May)
		2 = Kiremit (June to August)
16	Sex of child	0 = male
		1 = female
17	Twin pregnancy	0 = single birth
		1 = multiple birth

### Operational definition

*Very early neonatal mortality*—deaths of the newly born babies before 72 h of their postnatal age recorded in the data set.

*Additional feeding*—provision of anything other than breast milk (such as, formula milk plain water, sugar water).

*Risk fertility behavior*—those mothers who have High-risk fertility behaviors too young (under age 18) or too old (over age 34), short birth interval (less than 24 months after the preceding birth), and high parity (more than three children).

### Statistical method

When dealing with count data as the dependent variable, it is important to employ non-linear models that are based on non-normal distributions to describe the relationship with a group of predictor factors. Several widely used models for elucidating the association between an outcome variable and a set of explanatory variables in count data include Poisson regression, negative binomial regression, zero-inflated Poisson regression, zero-inflated negative binomial regression, and generalized Poisson regression models. These models are specifically designed to handle count data and offer effective means to analyze the relationship between the response variable and the predictors. By utilizing these models, researchers can accurately capture the complexities inherent in count data and gain valuable insights into the factors influencing the outcome variable.

### Poisson regression

When our outcome is a count variable that we can assume will follow a Poisson distribution around a predicted mean, Poisson regression is appropriate. The definition of Poisson regression is as follows:

$$\begin{aligned} & \log \left( {{ {\upmu }}\text{i} } \right) = \upbeta 0 + { {\upbeta }}1\;{\text{X}}1{\text{i}} + { {\upbeta }}2\;{\text{X}}2{\text{i}} + \cdots + { {\upbeta \text {k}}}\;{\text{Xki}} \\ &{\text{Yi}}\sim {\text{Poisson}}\left( {{ {{\upmu} \text {i}}}} \right) \\ \end{aligned}$$

where the symbol ~ means ‘is distributed as’ That is, we assume that our count outcome, *Yi*, has a Poisson distribution with mean *µi*, where *µi* depends on the values of *X*1, *X*2,… and their coefficients. The outcome is usually log transformed, since this often better represents the relationships between the outcome and the predictors, compared to a model without a transformation. Equivalently, we can write the Poisson model equation as

$${ {\upmu \text{i}}} = \exp \left( {{{\upbeta }}0 + { {\upbeta }}1\;{\text{X}}1{\text{i}} + { {\upbeta }}2\;{\text{X}}2{\text{i}} + \cdots + { {\upbeta \text{k}}}\;{\text{Xki}}} \right)$$

We derive this equation by exponentiating both sides of the previous equation. In Poisson regression, we assume that the observed numbers of events follow a Poisson distribution around the predicted mean. This has some interesting implications. In particular, recall the Poisson distribution’s property of equi-dispersion: the variance is equal to the mean. This implies that in a Poisson regression we expect the observations to have a variance equal to their predicted mean. use the method of maximum likelihood to find the coefficient estimates^11^.

### Negative binomial regression

When the assumption of equi-dispersion is not realistic – that is, if our outcome is over-dispersed a Poisson model may not be adequate. Instead, negative binomial regression model may best.

$$\begin{aligned} & \log \left( {\upmu {\text{i}}} \right) = \upbeta 0 + \upbeta 1{\text{ X1i}} + \upbeta 2{\text{ X2i}} + \cdots + \upbeta k{\text{Xki }} \\ & {\text{Yi}}\sim {\text{Neg}}\,Bin\left( \,{\mu{\text{i }},\upalpha \,} \right)\text{var} \left( {{\text{Yi }}} \right) = \upmu {\text{i }} + \upalpha \upmu i^{2} \\ \end{aligned}$$

This negative binomial model looks very similar to a Poisson model. Again, we are using a logarithmic transformation of the outcome. The difference is that here we are expecting the observed numbers of events to follow a negative binomial distribution, rather than a Poisson distribution. Negative binomial distribution has two parameters, *µ* and *α*. Usually, only the mean *µ* is expected to be related to the predictors, but the dispersion parameter *α* is also estimated.vi The model must specify how we expect *α* to relate to the variance. Here I choose to specify that var (Yi) = μi + αμi^2^. This is called the NB^2^-parameterisation. At any rate, in negative binomial regression the predicted variance var (*Yi*) depends on both *α* and the predicted mean. This implies that we expect observations around the predicted mean to be heteroscedastic^12^.

### Too many zeroes: zero-inflation

Sometimes we encounter situations where the count outcome we wish to model seems to follow a Poisson or a negative binomial distribution, except that the number of zeroes is much larger than expected under either of those models. In such a situation, statisticians say that the count variable has **excess zeroes**. Sometimes, a Poisson or negative binomial regression model can fit such data well this is the case if one of our covariates identifies a group of cases with a very low mean count, and if many of the zeroes come from this group. Then, although the outcome variable taken on its own seems to have excess zeroes, once the effects of the predictors have been taken into account, the distribution of counts around the predicted means follows a Poisson or negative binomial distribution well enough. However, there are many cases when a Poisson or negative binomial regression does not fit data with excess zeroes well. So, we need to consider models that are specifically designed for count data with excess zeroes. Two types of models are often used for this purpose: zero-inflated models and hurdle models^11^.

### Models for outcomes with excess zeroes

Modelling an outcome with excess zeroes is a little more complicated than an ordinary count model. Such models consist of two parts:

- One part predicts structural zeroes.
- The other part predicts the remaining counts.

This section will introduce zero-inflated and hurdle models for both Poisson and negative binomial count distributions. As we will see, in both zero-inflated and hurdle models, we use logistic regression to predict structural zeroes. But the zero-inflated and hurdle models differ in how we model the counts:

- In a zero-inflated model, the counts are modelled with a Poisson or negative binomial distribution, and predicted counts have a theoretical minimum of zero (because there can be sampling zeroes as well as structural zeroes).
- In a hurdle model, the counts are modelled with a zero-truncated Poisson or zero-truncated negative binomial distribution, and predicted counts have a theoretical minimum of 1 (because there can be no sampling zeroes). Mathematically, a zero-inflated Poisson model can be expressed as follows:
  $$\begin{aligned} & {\text{P}}\left( {{\text{Yi}} = 0} \right) = \pi {\text{i}} + \left( {1 - \pi {\text{i}}} \right){\text{e}} - \mu {\text{i}} \\ & {\text{P}}\left( {{\text{Yi}} = {\text{k}}} \right) = \left( {1 - \pi {\text{i}}} \right)\mu {\text{ike}} - \mu {\text{i}}/{\text{k}}!,{\text{k}} \ge 1 \\ \end{aligned}$$

The first equation describes the probability of observing zero events. This probability is the sum of the probability of a structural zero (*πi*) and the probability of a sampling zero [(1 − πi) e − μi]. The second equation describes the probability of observing 1, 2, 3,… or more events^11,13^.

### Generalized Poisson distribution (GPD)

Generalized Poisson distribution (GPD) was introduced by Consul and Jain as a limiting form of the generalized negative binomial (GNB) distribution. The GNB parameters are defined such that a Poisson distribution with two parameters is produced, in which one of them is as a dispersion parameter. With the additional of dispersion parameters, the GPD can accommodate overdispersion or under dispersion in the Poisson distribution^14^. Means and variance of Generalized Poisson distribution are E(Y) = µ and var(Y) = µ (1 + ά µ)^2^. If the value of ά is zero then the model formed is Poisson regression. If the value of ά greater than zero then it called over dispersion, while the value of ά less than zero then it called under dispersion. The method that used for estimate parameter of GPR model is Maximum Likelihood Estimation (MLE) with the combination of Newton–Raphson iteration^15^.

$${\text{Py}}({\text{Y}} = {\text{K}}) = \frac{{{\text{e}} - (\uplambda + \upalpha *{\text{k}})*(\uplambda + \upalpha *{\text{k}}){\text{k}} - 1}}{{{\text{k}}!}}$$

### Investigating over dispersion and model comparison

**Figure 1:**
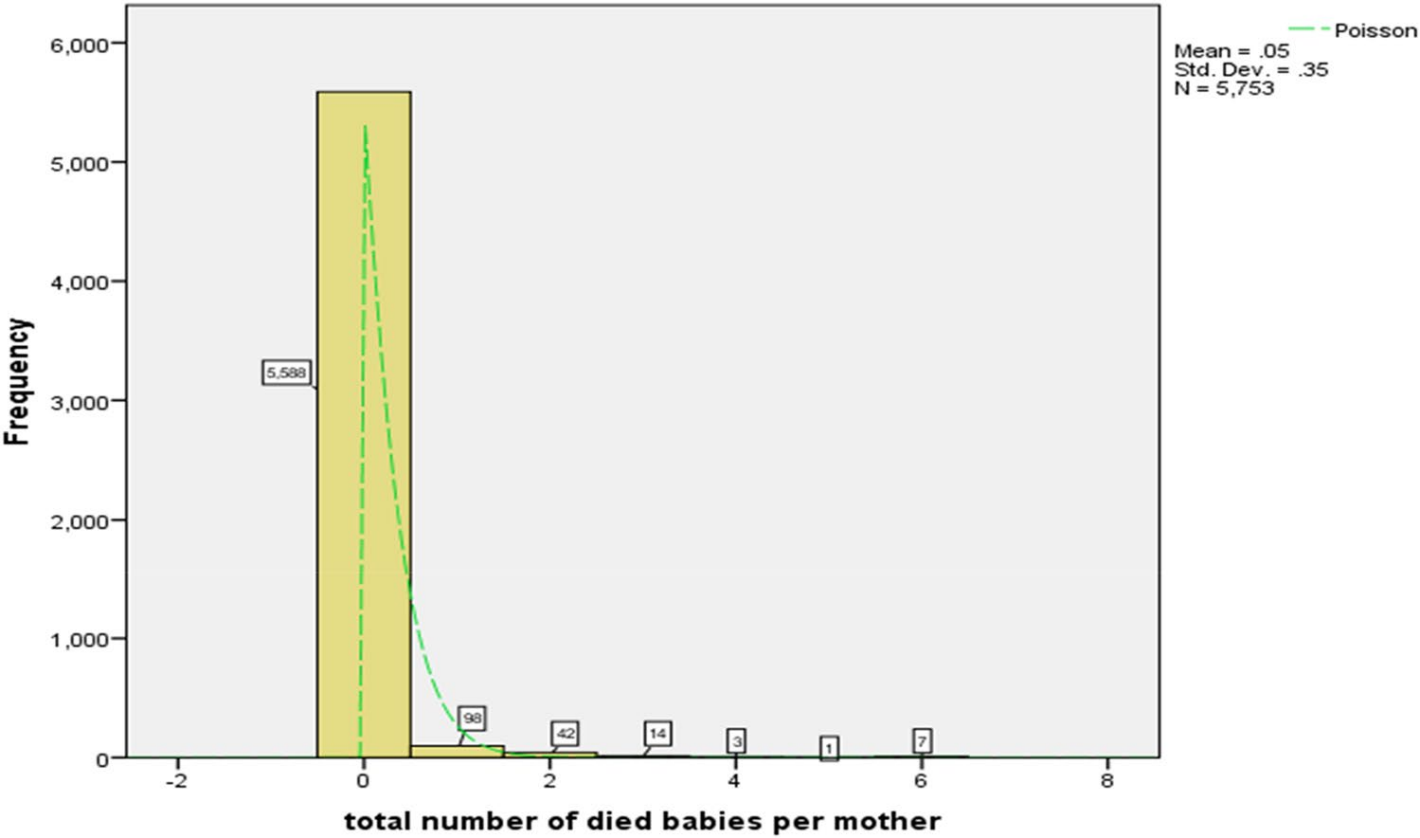
Histogram of the number of very early neonatal mortality of newly born babies per mother.

In Fig. 1, it is observed that the sample mean of the response variable, which represents the number of very early newborn infant deaths, is 0.05, while the sample variance is 0.1225 (standard deviation squared = 0.35). The fact that the mean is less extreme than the variance indicates an over dispersion situation. Furthermore, due to the presence of extra zeros in the data and the skewed nature of the data, it is expected that the Poisson model would not be suitable for accurately predicting the number of deaths.

To compare different models, the log likelihood, Akaike’s Information Criterion (AIC), and Bayesian Information Criterion (BIC) were utilized (reference 11). Based on the findings, the generalized Poisson regression model was determined to be the most appropriate count data model for this dataset (Table 2).

**Table 2:** compares various types of count data regression models.

S. no	Models	Log Pseudo likelihood (null)	Log Pseudo likelihood (model)	Akaike’s information criterion	Bayesian information criterion
1	Poisson	–	− 1197.5	2435.001	2568.15
2	Negative binomial	− 985.4717	− 939.3608	1920.722	2060.529
**3**	**Generalized poisson**	− **988.5794**	− **906.439**	**1854.878**	**1994.685**
4	Zero inflated poisson	− 976.751	− 930.3152	1914.63	2094.382
5	Zero inflated negative binomial	− 972.2449	− 933.7191	1923.438	2109.848

Bold values indicate best fitted count data model.

### Ethical approval

Ethical approval for this study was waived by ethical review board of Institute of Public Health, College of Medicine and Health Sciences, Wollo University and DHS International Program because all of the secondary data used in this study were collected from publicly available sources and lacked any personal information that might be used to identify specific people, groups, or study participants. Anonymity was used to ensure data confidentiality.

## Results

### Descriptive statistics

Among the 5527 weighted live births included in the study, 3.1% (n = 174) of newborns died within the first 72 h of life (Table 3). This represents over half (52.3%) of all neonatal deaths occurring within the five-year period covered by the survey. The proportion of very early neonatal mortality was slightly higher among males (2.4%) compared to females (2.2%). Additionally, nearly half (49.4%) of uneducated mothers experienced at least one very early neonatal death, and participants with risky fertility behaviors during pregnancy were more likely to have multiple deaths within the first 72 h of delivery (62.1%) (Table 3).

**Table 3:** General characteristics of died newborn with in the first 72 h of life.

S. no	Variables	Categories	At least 1 death per mother within 72 h n (%) (n = 174)
1	Region	Cities	36(21)
		Agriculture	53(30)
		Pastoralist	85(49)
2	Place of residency	Urban	52(30)
		Rural	121(70)
3	Wealth index	Average	73(14.6)
		Below average	75(43.4)
		Above average	25(41.8)
4	Number of births	Single birth	155(89)
		Twin birth	19(11)
5	Sex of babies	Male	95(54.6)
		female	79(45.4)
6	Antenatal care follow-up	No ANC visit	35(39.3)
		One to three visits	14(15.2)
		Four and above	41(45.5)
7	Place of delivery	Home	78(45)
		Public health institution	82(47)
		Private health institution	14(8)
8	Birth spacing in months	Less than 24 months	57(47.1)
		24 up to 36 months	24(19.9)
		Greater than 36 months	40(33)
9	Additional feeding initiation	No	141(81.4)
		Yes	32(18.6)
10	Marital status	Married	152(87.4)
		Unmarried	22(12.6)
11	Months of birth	Bega	43(24.7)
		Belg	77(44.3)
		kiremit	54(31)
12	Educational status	Not formal education	86(49.4)
		Primary education	72(41.4)
		Secondary education	5(2.8)
		Higher education	11(6.2)
13	Risky fertility behavior	Yes	108(62.1)
		No	66(37.9)
14	Respondent age	Mean (Sd)	28.6(± 6.4)

### Results of the generalized poisson regression analysis

Results in Table 4 provide estimates of the effect of some selected variables for very early mortality of newly born babies. Accordingly, among the predictors in the final model number of household member, multiple birth, taking additional feeding and higher birth order were the major predictors for mortality among newly born babies within the first seventy-two hours of their postnatal age per mother in Ethiopia. The expected number of very early neonatal deaths increased by a factor of 2.23 (IRR = 2.23; CI = 1.008, 4.916) for those birth order six and above compared to second to fifth birth order after controlling for other variables in the model. Similarly, expected number of mortalities in women who gave twin birth increased by a factor of 3.48 times compared to those gave single birth controlling for other variables in the model (IRR = 3.48; CI = 1.76, 6.887). These finding indicate that the risk of very early neonatal mortality decreases with each additional household member increment lived at the time of giving birth (IRR = 0.72; CI = 0.586, 0.885). Moreover, risk of early neonatal mortality for those babies got an additional feeding after birth was 77% less likely to die before seventy-two hours of age as compared to counterparts keeping other variables held constant in the model (IRR = 0.33; CI = 0.188, 0.579).

**Table 4:** Generalized Poisson regression analysis results of newly born babies died within the first seventy-two hours of their postnatal age per mother, Ethiopia [n = 174].

Variables	IRR	[95% confidence interval]	Robust Std. Err	*P*-value
Respondent age	1.02	0.967, 1.066	0.025	0.539
Number of household member	0.72	0.586, 0.885	0.076	0.002**
Place of residency (urban)				
Rural	0.90	0.408, 1.969	0.360	0.784
Wealth index (above average)				
Below average	0.58	0.277, 1.205	0.217	0.143
Average	0.82	0.397, 1.680	0.301	0.582
Twin child (single birth)				
Multiple birth	3.48	1.760, 6.887	1.212	0.000***
Sex of child (male)				
Female	0.89	0.563, 1.411	0.209	0.623
Place of delivery (public/gov’t sector)				
Home	1.26	0.728, 2.189	0.355	0.408
Private center	1.95	0.676, 5.625	1.054	0.217
Region (Cities)				
Agricultural	0.83	0.413, 1.674	0.297	0.605
Pastorals	1.69	0.818, 3.498	0.627	0.156
Additional feeding (No)				
Yes	0.33	0.188, 0.579	0.095	0.000***
Marital Status (married)				
Unmarried	1.49	0.697, 3.162	0.573	0.305
Educational Status (technical /higher education)				
Non educated	1.08	0.314, 3.714	0.681	0.904
Primary education	1.37	0.388, 4.838	0.882	0.626
Secondary education	0.30	0.054, 1.606	0.255	0.158
History of risk fertility (No)				
Yes	0.97	0.582, 1.612	0.252	0.902
Birth order (2nd–5th)				
First	0.94	0.520, 1.702	0.285	0.841
6th +	2.23	1.008, 4.916	0.900	0.048*
_cons	0.24	0.025, 2.314	0.277	0.217
Delta	0.41	0.325, 0.489	0.042	

Std. Error = Standard error.

* = significant at *P*-value < 0.05, ** = significant at *P*-value < 0.01, and *** = significant at *P*-value < 0.001. Bold values indicate best fitted count data model.

## Discussion

We conducted an investigation into the factors that contribute to the high mortality rate among newborn infants in Ethiopia within the first few days of life. Our findings revealed that several factors, including the number of household members, multiple births, additional feeding, and birth order, were associated with an increased risk of death. This study is the first of its kind to examine the determinants of mortality within the first three days of life in Ethiopia, providing valuable insights for policymakers to identify areas of improvement and track progress towards achieving the Millennium Development Goals (MDGs).

Our research confirmed that the risk of mortality within the first 72 h of life is higher for twins or multiple births compared to single births, which is consistent with previous studies^7,16–18^. Multiple births are often linked to premature birth, low birth weight, and biological immaturity, which increase the likelihood of adverse outcomes, including early newborn mortality^19^.

Additionally, birth order was found to be a significant predictor of infant death within the first 72 h of life, similar to findings from other studies^7,18,20,21^. The biological depletion hypothesis suggests that children born later may be less healthy than their older siblings due to being born to older mothers who have already given birth to multiple children and may be less physiologically capable of producing healthier offspring. However, there is evidence to suggest that first-born babies are also vulnerable to illness and death, as they are typically born to younger and less experienced mothers who may lack the necessary resources for adequate care^22,23^.

Furthermore, our study revealed that an increase in the size of the household was associated with a higher risk of very early neonatal mortality, consistent with previous findings^21,24,25^. This could be attributed to a reduction in parent–child emotional and physical attachments, which are crucial for promoting survival^23^. Additionally, larger family sizes may lead to food insecurity, resulting in poor maternal nutrition and adverse birth outcomes, including neonatal mortalities^26^.

Another significant factor associated with very early newborn mortality was the initiation of additional feeding. Our findings indicated that newborns who received additional feeding were less likely to die within the first three days of life compared to those who did not receive it. While breastfeeding is known to offer protection against mortality and disease, additional feeding may be beneficial in reducing very early neonatal deaths, particularly by preventing complications associated with hypoglycemia and hypothermia caused by delayed breastfeeding initiation and inadequate breast milk production in the initial days^27,28^.

In general, developing countries like Ethiopia tend to experience higher mortality rates among newborns within the first 72 h of life due to challenges in adapting to extra uterine life. Based on the 2019 mini-Ethiopian Demographic and Health Survey data, our study identified an increased number of household members, multiple births, additional feeding, and birth order six and above as significant predictors of mortality among newborns within the first 72 h of life per mother.

In conclusion, this study aimed to identify the key predictors of very early neonatal mortality using count regression models in Ethiopia. It utilized recent nationally representative data from the 2019 mini-Ethiopian Demographic and Health Survey. Out of 5527 weighted live births that occurred within five years preceding the survey, 174 (3.1%) experienced at least one very early neonatal mortality. These deaths accounted for 52.3% of all deaths among children under five. To reduce this high mortality rate, further efforts should focus on addressing the factors identified in this study, including the number of household members, multiple births, additional feeding, and higher birth order. Additionally, future studies should consider more detailed recommendations for practical interventions and strategies that can be implemented to reduce very early neonatal mortality in Ethiopia. Policymakers should carefully consider the policy implications of these findings and develop targeted approaches that address the specific needs of the population, taking into account socio-demographic characteristics, women’s empowerment, and partner education.

The strengths of this study lie in its use of a recent nationally representative dataset (2019 mini-EDHS) and the application of sampled weights and a well-fitted model (Generalized Poisson regression) to identify determinants of very early neonatal death in Ethiopia. However, it is important to note that this study relied on secondary data, and some variables were not included due to high rates of missing values, such as the number of antenatal visits, preceding birth interval, and breastfeeding initiation. As a cross-sectional survey, causality cannot be established, and there may be uncertainty regarding temporal associations.

## Data Availability

This study used 2019 mini EDHS child data set and extracted the outcome and explanatory variables. The datasets generated and analyzed in this study is not publicly available but can be obtained upon justifiable request from the corresponding author.

## Abbreviations

ANC: Antenatal care
C/S: Cesarian section delivery
CI: Confidence interval
EDHS: Ethiopia Demographic and Health Survey
EMDHS: Ethiopia Mini Demographic and Health Survey
ICF: International classification of functioning, disability, and health
MDGs: Millennium development goals
Sd: Standard deviation
SNNPR: Southern nation nationalities and peoples region
